# A Pilot Study: Extraction of a Neural Network and Feature Extraction of Generation and Reduction Mechanisms Due to Acute Stress

**DOI:** 10.3390/brainsci13030519

**Published:** 2023-03-21

**Authors:** Mi-Hyun Choi

**Affiliations:** Biomedical Engineering, Research Institute of Biomedical Engineering, School of ICT Convergence Engineering, College of Science & Technology, Konkuk University, Chungju 27478, Republic of Korea; kwjcc486@kku.ac.kr; Tel.: +82-43-840-3758

**Keywords:** functional connectivity, stress, recovery after stress, effect size

## Abstract

This study aimed to compare the functional connectivity (FC) assessed during acute stress and recovery after stress using the Montreal imaging stress task (MIST) in adults in their 20s and 30s with Korean Perceived Stress Scale (PSS) scores between 15 and 19 points inclusive. Four seed networks, including the salience network, default mode network, frontoparietal network, and dorsal attention network, were specified to extract the results. Healthy male and female adults who were required to make an effort to relieve stress were exposed to acute stress tasks, and the most common FCs were observed in the salience network, default mode network, and frontoparietal network during the stress and recovery phases. Compared to the stress phase, the increased effect size was significantly different in the recovery phase. In the stress phase, characteristically common FCs were observed in the dorsal attention network. During the recovery period, Salience network (Anterior Insula, R) and Salience network (anterior cingulate cortex, ACC)/Salience network (rostral prefrontal cortex, RPFC), Salience network (AInsula) and Salience network (RPFC), and Default Mode network (posterior cingulate) cortex, PCC) and fronto-parietal network (lateral prefrontal cortex, LPFC) FC were characteristically observed.

## 1. Introduction

Stress is a major risk factor for psychiatric disorders. There are many kinds of stress: acute, chronic, psychological, physical, and so on. Acute stress is a short-term response to a sudden, specific challenge or demand, such as a job interview, car accident, or public-speaking event. This type of stress is normal and often motivating, but can become problematic if it becomes chronic [1]. Chronic stress refers to long-term exposure to stressors, such as a difficult job, financial difficulties, or a traumatic life event. It can have a significant impact on physical and mental health, including increasing the risk of cardiovascular disease, depression, and anxiety [2]. Psychological stress refers to the mental and emotional strain caused by negative life events, such as a death in the family, relationship problems, or financial difficulties. This type of stress can result in emotional symptoms such as anxiety, depression, and irritability [3]. Physical stress refers to the demands placed on the body, such as injury, illness, or environmental factors such as extreme temperatures. This can lead to physical symptoms such as pain, fatigue, and reduced performance [4].

Studies have demonstrated the patterns of brain activity associated with stress [5]. Among them, functional connectivity (FC) refers to the correlation of the patterns of brain activity across different regions of the brain, typically measured using functional magnetic resonance imaging (fMRI) [6,7]. The study of functional connectivity in the context of acute stress has gained significant interest in recent years, as it provides valuable insights into the underlying neural mechanisms of stress and its effects on the brain [8,9].

Studies have shown that exposure to stress can lead to changes in FC within the brain, particularly in the amygdala and other regions involved in the stress response, such as the hypothalamus, insula, and anterior cingulate cortex. For example, studies have reported increased FC between the amygdala and hypothalamus during exposure to acute stress, suggesting that stress leads to a heightened emotional and physiological response [10,11]. Additionally, it has been reported that acute stress can lead to decreased FC between regions involved in executive control, such as the prefrontal cortex and insula, which can impair the ability to regulate emotions and manage stress [12,13,14,15].

By comparing the FC in the stress and recovery periods, researchers can identify the specific brain regions and connections that are affected by stress, and the mechanisms by which they recover. Furthermore, comparisons of FC during stress and recovery can help to identify potential compensatory mechanisms that may be involved in the recovery process, as well as potential targets for interventions aimed at improving the brain’s resilience to stress. In conclusion, comparing the FC in the stress and recovery periods is important for understanding the changes in brain activity that occur in response to stress, and for identifying potential targets for interventions to improve stress resilience.

This study aims to compare the FC assessed during acute stress and recovery after stress using the Montreal imaging stress task (MIST) in adults in their 20s and 30s with stress scores between 15 and 19 points inclusive.

## 2. Materials and Methods

### 2.1. Participants

In this study, 37 healthy adult men and women (24.4 ± 4.3 years), right-handed, with normal cognitive abilities, were tested. Recruited subjects ranged in age from 20 to 33 years, and were 20 females (24.6 ± 4.2 years) and 17 males (25.1 ± 3.0 years). To measure the stress level, Korean perceived stress scale (PSS) [16] was used before the intervention. Participants who had PSS scores of ≥15 and ≤19 were included in this study. When conducting magnetic resonance (MR) imaging, participants who had a pacemaker or metal fragments such as a metal pin in their body, which might affect the MR imaging, or who had claustrophobia were excluded from the study. Before the intervention, the participants were asked to refrain from smoking and drinking alcohol or coffee as that might affect brain activity, and the study purposes and contents were fully explained to them. Right-handedness was checked with the revised Edinburgh Reading Test [17]. The protocol for the research project was approved by the Institutional Review Committee of Konkuk University, where the work was undertaken, and the study conforms to the provisions of the Declaration of Helsinki (IRB number: 7001355-202010-HR-405).

### 2.2. Selection of Stress-Induced Task

The acute stress-induced task was presented using the MIST paradigm. MIST was designed by Dedovic et al. [5] to induce psychosocial stress. An investigator provides a negative feedback to a participant about their performance. Thus, the participant continuously receives negative feedback from others in an uncontrollable situation. Specifically, this induces social stress by making the participant aware of their poor performance compared to that of a virtual character. It is a commonly used task to induce stress.

To induce stress, the participants were asked to calculate the arithmetic operations by difficulty level with a time limit of 3 s, and whether the answers were correct and their average scores were provided. In addition, they were asked to maintain a pre-intervention total score of 95 points.

### 2.3. Intervention Design

**Figure 1:**
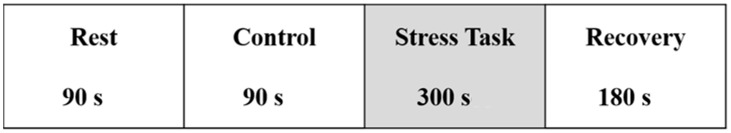
Experimental design.

The study design is presented in Figure 1. In the rest phase, the participant was asked to rest in a comfortable condition, while, in the control phase, the participant was asked to calculate arithmetic operations, without stress-inducing conditions. In the stress-task phase, the participant calculated the problem under a stress-inducing condition; the study set a time limit of 3 s using a timer to induce stress, and the participant’s average score and the results (correct, incorrect, and no response) were presented for 2 s as soon as they solved the problem, before moving on to the next question. Afterwards, in the recovery phase, the participant rested in a comfortable condition.

### 2.4. Functional MRI Acquisition

Images were scanned with a 3T MRI system (Magnetom TrioTim; Siemens Medical Systems, Erlangen, Germany) using a standard 16-channel head coil. Single-shot echo-planar fMRI scans were acquired in 29 continuous slices, parallel to the anterior commissure–posterior commissure line. The parameters for fMRI were: TR/TE, 2000/20 ms; FOV, 240 mm; flip angle, 77°; matrix, 128 × 128; slice thickness, 3 mm; and voxel size, 3.0 mm × 3.0 mm × 3.0 mm. Anatomical images were obtained using a T1-weighted 3D MPRAGE sequence with the following parameters: TR/TE, 1900/2.52 ms; FOV, 256 mm; flip angle, 9°; matrix, 256 × 256; slice thickness, 1 mm; and voxel size, 1.0 × 1.0 × 1.0 mm.

### 2.5. Functional Brain Imaging Analysis

The fMRI data were analyzed with the Statistical Parameter Mapping (SPM 12) software, version 12 (https://www.fil.ion.ucl.ac.uk/spm/software/spm12/; Wellcome Department of Cognitive Neurology, London, UK). All functional images were aligned with the anatomic images of the study using affine transformation routines built into the SPM 12 program. Time series of images acquired from the same participant were realigned using a least-squares approach and a 6-parameter (rigid body) spatial transformation. The first image in the list specified by the user was used as a reference to which all subsequent scans were realigned. The realigned scans were co-registered with the participant’s anatomic images obtained during each session and normalized to a template image in SPM 12, which uses the space defined by the Montreal Neurologic Institute. Motion correction was performed using a Sinc interpolation. Time-series data were filtered with a 240 s high-pass filter to remove artifacts due to cardiorespiratory and other cyclical influences. Additionally, the co-registered T1 and T2 images were used in a multichannel segmentation routine to extract probabilistic maps of six tissue classes: gray matter (GM), white matter (WM), cerebrospinal fluid (CSF), bone, soft tissue, and residual noise. The functional map was smoothened with an 8 mm isotropic Gaussian kernel before statistical analysis. Statistical analysis was performed at the group level using a general linear model and the theory of Gaussian random fields implemented in SPM 12.

To process the fMRI data, we used the functional connectivity (CONN) toolbox (https://web.conn-toolbox.org/), which was implemented in the SPM 12 software. ROI-to-ROI analysis was conducted to examine which FC was implicated in inter-individual variations using the CONN toolbox. We predefined 105 ROIs in the CONN toolbox based on the Harvard–Oxford cortical and subcortical structural atlases (https://neurovault.org/collections/262/). The brainstem and cerebellum were excluded. We selected statistically significant ROI-to-ROI connections that correlated with the performance of the recognition tests by cluster-level inferences based on spatial pairwise clustering statistics (SPC) [18] with default settings in CONN. The T-statistics of the entire ROI-to-ROI matrix were estimated using a general linear model. Four networks were used as seeds, and 11 ROIs were selected. The default mode network (DMN) includes the medial prefrontal cortex (MPFC), lateral parietal lobe (LP), and posterior cingulate cortex (PCC); and the salience network (SN) includes the anterior cingulate cortex (ACC), anterior insular cortex (A Insula), rostral prefrontal cortex (RPFC), and supramarginal gyrus (SMG). The dorsal attention network (DAN) includes frontal eye fields (FEF) and the intraparietal sulcus (IPS), while the frontoparietal network includes the lateral prefrontal cortex (LPFC) and posterior cingulate cortex (PCC). The statistical parametric map was thresholded using a significance level of *p* < 0.01 (uncorrected). The resulting suprathreshold areas defined a series of non-overlapping clusters. Cluster-level FDR-corrected *p* < 0.05 was applied.

The effect size of FC, which was common in the stress phase and the recovery phase, was extracted, and paired *t*-test (IBM SPSS Statistics 27) was performed to examine whether the effect size differs by phase.

## 3. Results

**Figure 2:**
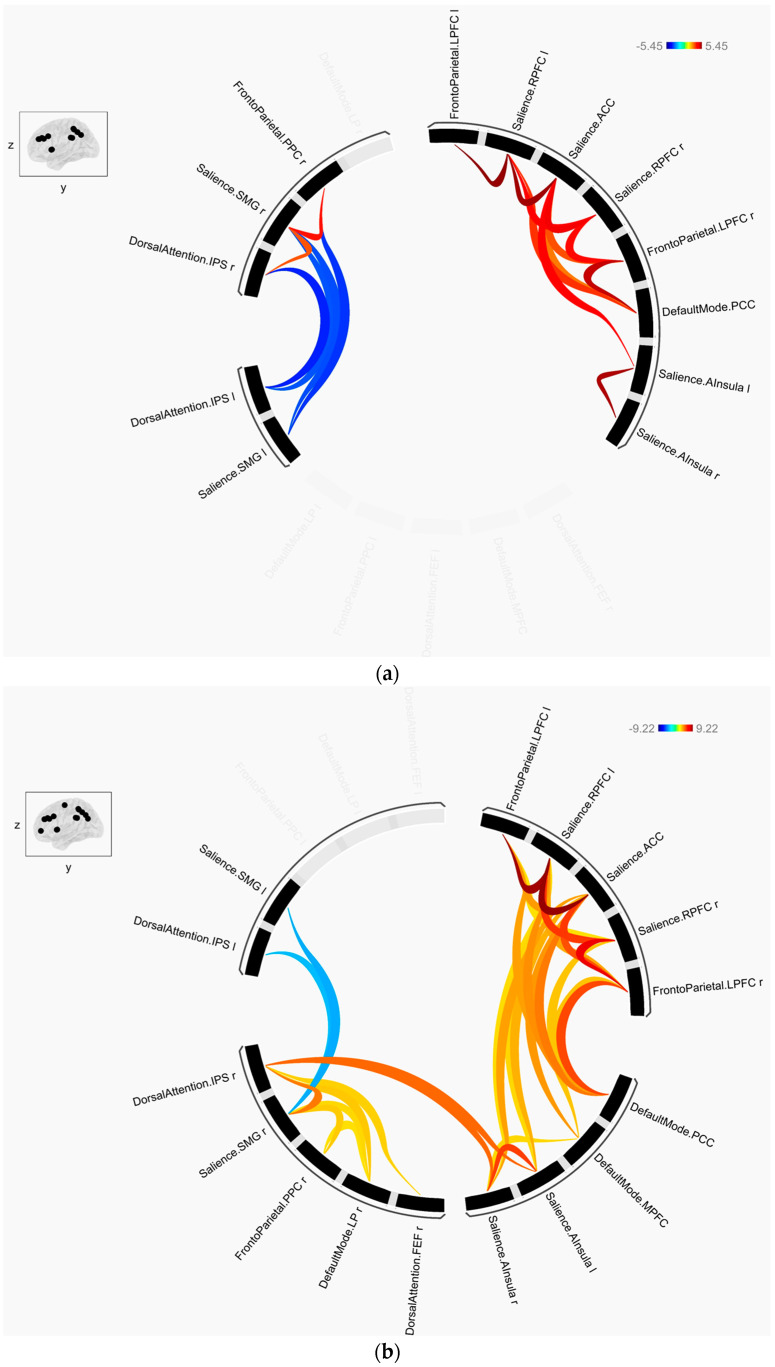
ROI-to-ROI connectivity map of the (a) stress and (b) recovery phases.

FC was obtained from the data measured during the stress and recovery phases (Table 1 and Table 2, Figure 2). FCs that were commonly shown in both phases were as follows: SN (RPFC, Left) and frontoparietal network (FPN) (LPFC, L), SN (ACC) and SN (RPFC, L), DMN (PCC) and FPN (LPFC, Right), SN (RPFC, R) and SN (ACC)/RPFC, L), SN (A Insula, L) and SN (ACC), FPN (LPFC, R) and SN (RPFC, R/ACC), and DMN (PCC) and SN (RPFC, L). Among the commonly shown FCs, negative t-values were observed within the SN (SMG, L; and SMG, R) and between the DAN (IPS, L) and SN (SMG, R).

**Figure 3:**
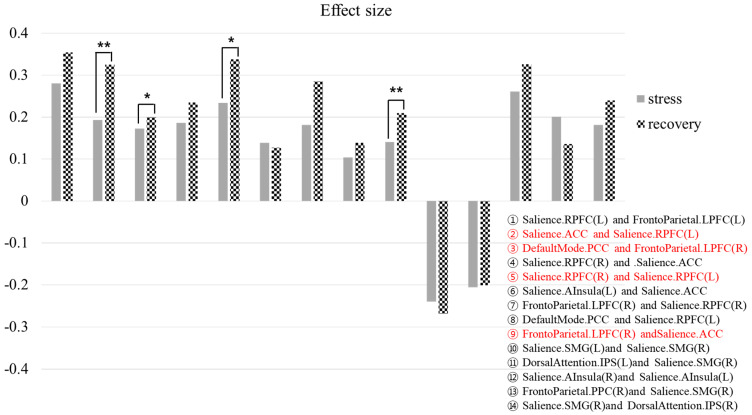
Comparison of the effect sizes of common functional connectivity in the stress phase and recovery phases. Red color : FC with statistically significant difference (** *p* < 0.01, * *p* < 0.05).

Figure 3 compares the effect sizes of the common FCs. Several networks in the recovery phase showed a greater significant difference in the increased FCs compared to those in the stress phase. These included the SN (ACC) and SN (RPFC, L) (*p* = 0.01), DMN (PCC) and FPN (LPFC, R) (*p* = 0.035), SN (RPFC, R) and SN (RPFC, L) (*p* = 0.045), and FPN (LPFC, R) and SN (ACC) (*p* = 0.008).

FCs that were specifically observed in the stress phase included the DMN (PCC) and SN (ACC), DAN (IPS, L) and DAN (IPS, R), and SN (SMG, L) and FPN (PCC, R).

In the recovery phase, connectivity between the SN (ACC) and FPN (LPFC, L), SN (A Insula, R) and SN (ACC)/RPFC, L), SN (A Insula, L) and SN (RPFC, L), and SN (RPFC, R) and frontoparietal network (LPFC, L) was observed, while FC of FPN (LPFC, R) and SN (RPFC, L), was observed. FC of the DMN (MPFC) and SN (ACC)/FPN (LPFC, R), and DMN (PCC) and FPN (LPFC, L)/SN (RPFC, R) was observed.

## 4. Discussion

The DMN, SN, DAN, and FPN are four different brain networks that have been implicated in the regulation of stress. The DMN is a network of brain regions that is active during rest and deactivated during task performance. Acute stress has been found to increase FC within the DMN and decrease FC between the DMN and task-positive network (TPN), which is a network of brain regions that is active during task performance [19,20]. These changes are thought to reflect increased rumination and decreased attentional control after stress. The SN includes brain regions that are involved in the detection and integration of information from various sensory modalities and the regulation of attention. Acute stress has been found to increase FC within the SN, especially between the anterior insula, which is involved in the processing of interoceptive signals, and the ACC, which is involved in executive control and attention [21]. The DAN includes brain regions that are involved in attention and sensorimotor processing [22,23,24,25,26]. Acute stress has been found to decrease FC within the DAN, especially between the dorsal frontal cortex, which is involved in attention, and the parietal cortex, which is involved in spatial processing [7,8]. The FPN includes brain regions that are involved in attention, executive control, and working memory [27,28,29,30]. Acute stress has been found to increase FC within the FPN, especially between the lateral frontal cortex, which is involved in executive control, and the parietal cortex [9,19]. It is worth noting that, while these findings suggest that different brain networks are involved in stress regulation, the exact role and mechanisms of these networks in stress remain unclear and are the subject of ongoing research.

Studies have found that acute stress leads to alterations in FC in various brain networks, including the DMN, SN, and central executive network [9,31]. These changes are thought to reflect the recruitment of brain regions involved in stress regulation and the disruption of cognitive processes. For example, studies have found that acute stress increases FC between the amygdala, which is involved in the stress response, and the ACC [11]. During stress, resources are redirected toward regions involved in detecting salient stimuli (ventral attention network; VAN) at the cost of executive functioning (FPN) [32], potentially accompanied by increased DMN activity [33], DMN being a network involved in self-referential processing. During stress recovery, there is a distinct resource reallocation from the acute phase, with the increase in VAN and the decrease in FPN resources being roughly reversed [32]. Additionally, acute stress has been found to decrease FC between the DMN and TPN. These changes are thought to reflect decreased attentional control and increased rumination after stress.

This study compared the effect sizes of common FCs in the stress and recovery phases, and showed that the effect sizes of the connection within the SN (ACC and RPFC, L; RPFC, R and RPFC, L), and between the DMN (PCC) and FPN (LPFC, R), and FPN (LPFC, R) and SN (ACC) increased during the recovery phase compared to the stress phase. This suggests that the FC between these brain regions (SN, DMN, and FPN) during the rest period after stress-inducing tasks may be stronger compared to that during the tasks themselves. This increased FC could indicate a heightened state of recovery and restoration in the brain after stress.

This study demonstrated that when stress was induced, FCs between the DMN (PCC) and SN (ACC), within the DAN (IPS, L and IPS, R), and between the SN (SMG, L) and FPN (PCC, R) were specifically observed. In terms of the FC between the DMN and SN during stress, some studies have found that the connectivity between the PCC, a key node in the DMN, and the ACC, a key node in the SN, increases during stress, while others have found that this connectivity decreases. In terms of the FC between the DAN and SN, some studies have found that the connectivity between the IPS in the DAN and the SMG in the SN increases during stress, while others have found that it decreases. When considering the FC between the FPN and SN, some studies have found that the connectivity between the PCC in the FPN and the SMG in the SN increases during stress, while others have found that it decreases. In conclusion, the FC between different brain networks during stress-inducing tasks is still an active area of research, and the results are not yet consistent across studies. Further research is needed to fully understand the underlying mechanisms and to determine the generalizability of these findings.

Studies have found that performing MIST leads to alterations in FC in the DMN, which is a network of brain regions that is active during rest and deactivated during task performance. Specifically, MIST has been found to increase FC within the DMN and decrease the same between the DMN and TPN [34]. These changes are thought to reflect increased rumination and decreased attentional control after stress. Additionally, several studies have found that FC in the DMN and TPN recovers to baseline levels after the stress-inducing task. However, other studies have reported that, especially in individuals with high levels of stress or anxiety, it takes longer for FC to recover.

Healthy adults who are required to make an effort to relieve stress were exposed to acute stress tasks and the most common FCs were observed in the SN, DMN, and FPN during the stress and recovery phases. Compared to the stress phase, the increased effect size was significantly different in the recovery phase. In the stress phase, characteristically common FCs were observed in the DAN. During the recovery phase, FCs were observed within the SN (A Insula, R and ACC)/ RPFC; A Insula and RPFC), and between the DMN (PCC) and FPN (LPFC). Compared to previous studies, our findings suggest a complex network of interactions between different brain regions during the post-stress recovery phase. The FC between the SN and FPN suggests a role for these regions in modulating the effects of stress [35]. Similarly, the FC between the DMN and various regions of the SN and FPN may play a role in regulating the stress response. It is important to note that these findings are from the preliminary stages and need to be further explored in larger and more diverse samples to gain a better understanding of their meaning and implications.

The comparison of FC in the stress and recovery phases is important because it provides insight into the changes in brain function that occur as a response to stress, and how the brain returns to a state of homeostasis during recovery. The common FC patterns in both phases suggest the presence of similar neural networks involved in the stress and recovery processes. The differences in FC between the stress and recovery phases, such as the decrease in the within-DAN (IPS, L-IPS, R) and SAN (SMG, L)-FPN (PCC, R) connectivity in the stress phase and the emergence of new functional connections in the recovery phase, suggest the presence of dynamic changes in brain function as a response to stress and recovery.

## 5. Conclusions

These findings have implications for the understanding of stress-related disorders and the development of potential treatments. For example, changes in FC in the stress and recovery phases may be used as markers of stress-related disorders and their severity, and interventions that target specific FC patterns may be used to alleviate symptoms and improve recovery.

There are several limitations to this study that should be considered. First, the sample size of the study is relatively small, which may limit the generalizability of the results to other populations. Second, the study only included individuals who experienced acute stress, which may not fully capture the effects of chronic stress or stress experienced in different contexts. Additionally, the exclusion and inclusion criteria of the study were not well-defined, which may have introduced confounding factors into the analysis.

A study on the differences in brain neural mechanisms during stress and recovery after stress through a comparative study between a normal group and a group with a high-stress index (e.g., chronic-stress group) is needed. In addition, in stress studies, considering the effects of many comorbid factors (mental disorders, smoking, alcohol intake, and drug use), it is thought that further research is needed to extract conclusions that can generalize the neural mechanisms of the brain caused by stress.

## Figures and Tables

**Table 1:** ROI-to-ROI functional connectivity statistics of the stress phase.

Analysis Unit				
Cluster 1/9	Mass = 371.47	p-unc	p-FDR	p-FWE
networks.Salience.RPFC (L) (−32,45,27)–networks.FrontoParietal.LPFC (L) (−43,33,28)		T(36) = 5.45	0.000004	0.000467
networks.Salience.ACC (0,22,35)–networks.Salience.RPFC (L) (−32,45,27)		T(36) = 5.32	0.000006	0.000467
networks.DefaultMode.PCC (1,−61,38)–networks.FrontoParietal.LPFC (R) (41,38,30)		T(36) = 4.96	0.000017	0.000715
networks.Salience.RPFC (R) (32,46,27)–networks.Salience.ACC (0,22,35)		T(36) = 4.55	0.000058	0.001986
networks.Salience.RPFC (R) (32,46,27)–networks.Salience.RPFC (L) (−32,45,27)		T(36) = 4.32	0.000117	0.003341
networks.Salience.AInsula (L) (−44,13,1)–networks.Salience.ACC (0,22,35)		T(36) = 4.15	0.000197	0.004805
networks.FrontoParietal.LPFC (R) (41,38,30)–networks.Salience.RPFC^®^ (32,46,27)		T(36) = 3.91	0.00039	0.008331
networks.DefaultMode.PCC (1,−61,38)–networks.Salience.RPFC (L) (−32,45,27)		T(36) = 3.53	0.001156	0.016535
networks.FrontoParietal.LPFC (R) (41,38,30)–networks.Salience.ACC (0,22,35)		T(36) = 3.26	0.00244	0.02782
networks.DefaultMode.PCC (1,−61,38)–networks.Salience.ACC (0,22,35)		T(36) = 2.83	0.07501	0.063607
Cluster 2/9	Mass = 94.44	p-unc	p-FDR	p-FWE
networks.DorsalAttention.IPS (L) (−39,−43,52)–networks.DorsalAttention.IPS (R) (39,−42,54)		T(36) = −3.72	0.000679	0.011612
networks.Salience.SMG (L) (−60,−39,31)–networks.FrontoParietal.PPC (R)(52,−52,45)		T(36) = −3.53	0.00116	0.016535
networks.Salience.SMG (L) (−60,−39,31)–networks.Salience.SMG (R) (62,−35,32)		T(36) = −3.28	0.002335	0.02782
networks.DorsalAttention.IPS (L) (−39,−43,52)–networks.Salience.SMG (R)(62,−35,32)		T(36) = −3.19	0.002913	0.029298
Cluster 3/9	Mass = 54.03	p-unc	p-FDR	p-FWE
networks.Salience.AInsula (R) (47,14,0)–networks.Salience.AInsula (L) (−44,13,1)		T(36) = 5.20	0.000008	0.000467
Cluster 4/9	Mass = 49.79	p-unc	p-FDR	p-FWE
networks.FrontoParietal.PPC (R) (52,−52,45)–networks.Salience.SMG (R) (62,−35,32)		T(36) = 3.83	0.000492	0.009342
networks.Salience.SMG (R) (62,−35,32)–networks.DorsalAttention.IPS (R)(39,−42,54)		T(36) = 3.20	0.0029	0.029298

**Table 2:** ROI-to-ROI functional connectivity statistics of the recovery phase.

Analysis Unit				
Cluster 1/9	Mass = 1090.39	p-unc	p-FDR	p-FWE
networks.Salience.ACC (0,22,35)–networks.Salience.RPFC (L) (−32,45,27)		T(36) = 9.22	0	0
networks.Salience.RPFC (L) (−32,45,27)–networks.FrontoParietal.LPFC (L)(−43,33,28)		T(36) = 9.04	0	0
networks.FrontoParietal.LPFC (R) (41,38,30)–networks.Salience.RPFC (R) (32,46,27)		T(36) = 7.76	0	0
networks.Salience.RPFC (R) (32,46,27)–networks.Salience.RPFC (L) (−32,45,27)		T(36) = 5.90	0.000001	0.000039
networks.Salience.RPFC (R) (32,46,27)–networks.Salience.ACC (0,22,35)		T(36) = 5.83	0.000001	0.000039
networks.DefaultMode.PCC (1,−61,38)–networks.FrontoParietal.LPFC (R) (41,38,30)		T(36) = 5.62	0.000002	0.000055
networks.FrontoParietal.LPFC (R) (41,38,30)–networks.Salience.ACC (0,22,35)		T(36) = 5.51	0.000003	0.000067
networks.DefaultMode.PCC (1,−61,38)–networks.Salience.ACC (0,22,35)		T(36) = 4.78	0.000029	0.000418
networks.DefaultMode.PCC (1,−61,38)–networks.Salience.RPFC (L) (−32,45,27)		T(36) = 4.32	0.000117	0.001542
networks.DefaultMode.MPFC (1,55,−3)–networks.Salience.ACC (0,22,35)		T(36) = 4.21	0.000162	0.001843
networks.Salience.ACC (0,22,35)–networks.FrontoParietal.LPFC (L) (−43,33,28)		T(36) = 4.10	0.000227	0.00243
networks.Salience.AInsula (R) (47,14,0)–networks.Salience.ACC (0,22,35)		T(36) = 4.06	0.000251	0.002472
networks.DefaultMode.PCC (1,−61,38)–networks.FrontoParietal.LPFC (L) (−43,33,28)		T(36) = 4.05	0.00026	0.002472
networks.FrontoParietal.LPFC (R) (41,38,30)–networks.Salience.RPFC (L) (−32,45,27)		T(36) = 3.92	0.000383	0.003449
networks.Salience.AInsula (L) (−44,13,1)–networks.Salience.ACC (0,22,35)		T(36) = 3.74	0.000636	0.005181
networks.DefaultMode.PCC (1,−61,38)–networks.Salience.RPFC (R) (32,46,27)		T(36) = 3.40	0.001651	0.011763
networks.DefaultMode.MPFC (1,55,−3)–networks.FrontoParietal.LPFC (R) (41,38,30)		T(36) = 3.35	0.001892	0.012943
networks.Salience.AInsula (L) (−44,13,1)–networks.Salience.RPFC (L) (−32,45,27)		T(36) = 3.05	0.004267	0.02516
networks.Salience.AInsula (R) (47,14,0)–networks.Salience.RPFC (L) (−32,45,27)		T(36) = 2.94	0.005704	0.029555
networks.Salience.RPFC (R) (32,46,27)–networks.FrontoParietal.LPFC (L) (−43,33,28)		T(36) = 2.90	0.006269	0.031146
Cluster 2/9	Mass = 245.44	p-unc	p-FDR	p-FWE
networks.DorsalAttention.IPS (R) (39,−42,54)–networks.Salience.AInsula (L) (−44,13,1)		T(36) = 5.08	0.000012	0.000215
networks.DorsalAttention.IPS (R) (39,−42,54)–networks.Salience.AInsula (R) (47,14,0)		T(36) = 5.06	0.000013	0.000215
networks.DorsalAttention.IPS (R) (39,−42,54)–networks.Salience.SMG (R) (62,−35,32)		T(36) = 4.78	0.000029	0.000418
networks.DorsalAttention.IPS (R) (39,−42,54)–networks.DefaultMode.LP (R)(47,−67,29)		T(36) = 3.28	0.002314	0.015221
networks.Salience.SMG (R) (62,−35,32)–networks.FrontoParietal.PPC (R) (52,−52,45)		T(36) = 3.23	0.002628	0.016644
networks.DorsalAttention.IPS (R) (39,−42,54)–networks.DorsalAttention.FEF (R) (30,−6,64)		T(36) =3.14	0.00336	0.020522
networks.Salience.SMG (R) (62,−35,32)–networks.DefaultMode.LP (R) (47,−67,29)		T(36) = 3.01	0.004742	0.02534
networks.FrontoParietal.PPC (R) (52,−52,45)–networks.DefaultMode.LP (R)(47,−67,29)		T(36) = 2.89	0.006454	0.031146
Cluster 3/9	Mass = 84.80	p-unc	p-FDR	p-FWE
networks.Salience.AInsula (R) (47,14,0)–networks.Salience.AInsula (L) (−44,13,1)		T(36) = 5.76	0.000001	0.000042
networks.Salience.AInsula (R) (47,14,0)–networks.DefaultMode.MPFC (1,55,−3)		T(36) = 3.04	0.004417	0.025177
Cluster 4/9	Mass = 54.64	p-unc	p-FDR	p-FWE
networks.Salience.SMG (R) (62,−35,32)–networks.Salience.SMG (L) (−60,−39,31)		T(36) = −3.85	0.000468	0.003998
networks.Salience.SMG (R) (62,−35,32)–networks.DorsalAttention.IPS (L) (−39,−43,52)		T(36) = −3.54	0.001137	0.008452

## Data Availability

The datasets generated for this study are available upon request from the corresponding author.
